# Prevalence and characteristics of hearing and vision loss in preschool children from low income South African communities: results of a screening program of 10,390 children

**DOI:** 10.1186/s12887-021-03095-z

**Published:** 2022-01-05

**Authors:** Susan Eksteen, Robert H. Eikelboom, Hannah Kuper, Stefan Launer, De Wet Swanepoel

**Affiliations:** 1 Department of Speech-Language Pathology and Audiology, University of Pretoria, Lynnwood Rd, Hatfield, Pretoria, Gauteng South Africa; 2 Ear Science Institute Australia, Subiaco, Australia; 3 Ear Sciences Centre, The University of Western Australia, Nedlands, Australia; 4 International Centre for Eye Health, Clinical Research Department, London School of Hygiene and Tropical Medicine (LSHTM), London, England; 5 Sonova AG, Science & Technology, Stäfa, Switzerland; 6 School of Health and Rehabilitation Science, University of Queensland, Brisbane, Australia

**Keywords:** Hearing loss, Vision loss, Preschool children, Low- and middle-income community, mHealth

## Abstract

**Background:**

The majority of children with sensory impairments live in low- and middle-income countries. More studies of hearing and vision impairment prevalence are needed, in order to generate more accurate estimates of trends in sensory impairments. This study aimed to estimate the prevalence and describe the characteristics of hearing and vision loss among preschool children (4–7 years) in an underserved South African community following community-based mobile health (mHealth) supported hearing and vision services.

**Methods:**

A screening program of sensory impairments was undertaken of children attending preschools in the communities of Khayelitsha and Mitchell’s Plain, Cape Town, from September 2017 until June 2019. Hearing and vision screening were done by trained community health workers using mHealth technology. Children who failed hearing and vision screening were seen for follow-up assessments at their preschools. Follow-up assessments were conducted using smartphones that host point-of-care validated and calibrated hearing and vision testing applications (hearTest app, hearX Group, South Africa and PeekAcuity app, Peek Vision, United Kingdom). Descriptive statistical analysis and logistic regression analysis were conducted after extracting data from a secure cloud-based server (mHealth Studio, hearX Group) to Microsoft Excel (2016).

**Results:**

A total of 10,390 children were screened at 298 preschools over 22 months. Of the children screened, 5.6 and 4.4% of children failed hearing and vision screening respectively. Community-based follow-up hearing tests were done at the preschools on 88.5% (514) of children of whom 240 children (54.2% female) presented with hearing loss. A preschool-based follow-up vision test was done on 400 children (88.1%). A total of 232 children (46.1% female) had a vision impairment, and a further 32 children passed the test but had obvious signs of ocular morbidity. Logistic regression analysis found that age was a significant predictor of vision loss (*p* < 0.05), but not for hearing loss (*p* = 0.06). Gender was not a significant predictor of hearing (*p* = 0.22) or vision loss (*p* = 0.20).

**Conclusions:**

Hearing loss is prevalent in at least 22 per 1000 and vision loss in at least 23 per 1000 preschool children in an underserved South African community*.* Timely identification of sensory losses can be facilitated through community-based hearing and vision services supported by mHealth technology.

## Background

Childhood hearing and vision loss are significant contributors to the global burden of disease [1, 2] affecting 38.7 and 32.5 million children under 10 years, respectively [2]. According to the World Health Organization (WHO), the majority of childhood hearing loss (60%) and vision loss (80%) can be treated or prevented if identified early [3, 4]. Therefore, periodic hearing and vision screening are considered integral strategies for preventative paediatric health care [5–8]. Early detection of sensory impairments is essential for facilitating early childhood development, socioemotional well-being and academic success, [1, 9–12] as well as the sustainable development goals (SDGs) related to education [1, 2, 13]. Early-childhood screening in preschools can identify children with congenital sensory losses, as well as those with late-onset, progressive, or fluctuating hearing and vision loss, thus facilitating intervention prior to school entry [6, 12, 14–16].

Unfortunately, the majority of children (80 to 90%) with sensory impairments live in low- and middle-income countries (LMICs) [2, 4, 10, 12] where services are usually unavailable or inaccessible, because of an absence of systematic screening programmes for children, prohibitive equipment cost and a shortage of trained personnel [11, 17, 18]. The prevalence of hearing and vision loss for children aged between 5 and 9 years are estimated at 4.5 and 3.1% respectively in sub-Saharan Africa in contrast to 2.2 and 1.3% respectively in high-income North America, demonstrating the need for attention to sensory impairment in LMICs [2]. Most cases of childhood hearing and vision loss have preventable causes that are common in low-to-middle-income countries (LMICs) and is often related either to infection or nutrition [8–10, 16, 19]. Unfortunately, children with disabilities in LMICs have considerably limited access to non-emergency health resources [11, 19] and are therefore prone to be left behind under the SDGs era without timely and appropriate intervention from early childhood [2, 16, 20].

Estimating the prevalence of sensory loss in this population is an important step to ensure adequate planning and successful implementation of community-based hearing and vision care in preschools in this context. There is a lack of contemporary population-based information about childhood hearing loss and visual impairment, from which the scope and priorities for prevention and treatment can be identified [1, 2, 6, 10, 12, 21, 22]. Particularly in high-burden LMICs, where these disabling conditions are highly prevalent, more studies of hearing and vision impairment prevalence are needed, in order to generate more accurate estimates of trends in sensory impairments [1, 2, 10]. Until recently, these surveys have been complex to undertake, relying on expensive equipment and trained staff, explaining the lack of data. The past few years have seen a rapid expansion of the evidence base on the value of community-based programmes incorporating non-professionals using solutions based on smartphone and internet technologies (mobile health (mHealth) technology) for hearing and vision services [15, 21, 23–30]. A South African study by Eksteen et al., (2019) recently reported the first hearing and vision screening for preschool children using smartphone-based technologies [26]*.* In this study, trained community health workers (CHWs) used validated smartphone-based applications (apps) for hearing screening (hearScreen app; hearX Group, South Africa) [21, 23, 24, 26–28, 30] and vision screening (Peek Acuity app; peekVision, United Kingdom) [15, 25]. In order to overcome loss to follow-up previously shown to affect the outcomes of screening programmes [8, 23, 24, 31], the study included a community-based first-line follow-up assessment for those who failed screening by also utilizing validated mHealth technology [26].

The aim of this study was to estimate the prevalence and describe the characteristics of hearing and vision loss among preschool children (4–7 years) in an underserved South African community following the mHealth supported community-based hearing and vision services described by Eksteen et al., (2019).

## Methods

Institutional Review Board clearance for the study was obtained from the University of Pretoria (HUM020/1019).

### Context and population

A community-based hearing and vision screening program for preschool children by community health workers (CHWs) was implemented using validated mHealth technologies [26, 32]. Four non-professionals from the community, none who had previous training in hearing or vision healthcare, were appointed and trained as CHWs to conduct the hearing and vision screening of all children included in the study at their preschools of the partially informal townships of Khayelitsha and Mitchell's Plain in South Africa [26, 32]. This program was undertaken from September 2017 to June 2019. The majority (97.1%; (181,145/186803)) of households within Khayelitsha and Mitchell's Plain are classified as low- and middle-income and the population of children aged 5 to 9 years was estimated as 61,094 in 2011 [33]. All children between the ages of 4 and 7 years attending preschools in the targeted areas for whom consent was obtained, received hearing and vision screening tests [26]. Children who failed either test had a follow-up assessment at their preschool [26]. If indicated, children were referred to their nearest clinic for intervention. This study estimated the prevalence of hearing and vision loss, based upon the results of the follow-up assessment.

#### Initial screening for hearing and vision

Hearing and vision screening were done by trained CHWs at the preschools in the community using smartphones that host point-of-care validated hearing and vision screening applications (hearScreen app, hearX Group, South Africa and Peek Acuity app, Peek Vision, United Kingdom) [26]. The hearScreen app is a low-cost app operable on an entry-level smartphone running Android OS software with off-the-shelf calibrated circumaural headphones that utilises pre-specified screening protocols to assess hearing using automated sequences and employs noise-monitoring algorithms for quality control [21, 23, 24, 34–37]. The peekAcuity app was designed and validated to test visual acuity proved capable of accurate and repeatable acuity measurements [15, 25]. The use of these validated smartphone screening apps incorporating automated testing and measures of quality control allowed trained CHWs to decentralise hearing and vision screening and to identify cases for referral [21, 23–25, 27, 29, 34–37]. A detailed description of the pilot and preparation phase of the programme, the training for CHWs, screening procedures and equipment were previously described by Eksteen et al. [26, 32]. Thresholds for failing the hearing screening were set at 25 dB hearing level at 1, 2 and 4 kHz from September 2017 until December 2018, and 30 dB HL at 1 kHz and 25 dB HL at 2 and 4 kHz from January to June 2019 [32]. Children were considered to have failed the initial vision screening if they had a visual acuity of less than 0.3 LogMAR in both eyes, or less than 0.4 LogMAR in one eye regardless of acuity in the other eye [4].

#### Follow-up assessments

All children who failed the screening were scheduled to undergo a follow-up assessment at their preschool.

Children who failed the hearing test received a follow-up assessment by an Audiologist at their preschool a week or two later [32]. The follow-up hearing assessment included otoscopy (Welch Allyn otoscope) and air conduction threshold pure tone audiometry using the validated hearTest app (hearX Group, South Africa) [32, 34, 35] on a Samsung A3 smartphone with the operating system Android version 8.0 (Google, United States of America), connected to supra-aural Sennheiser HD280 headphones (Sennheiser, Wedemark, Germany). Equipment had been calibrated according to prescribed standards (International Organization for Standardization, ISO 389–1). The app is calibrated to monitor environmental noise with the smartphone microphone [23, 35–37]. A warning was given when environmental noise exceeded minimal permissible ambient noise levels and the test could be paused until the noise levels were within an acceptable range [32]. Automated audiometry consisted of air conduction testing at 0.5 to 8 kHz starting at an intensity level of 40 dB HL until a minimum response level of 10 dB HL [32]. The threshold determination sequence follows the Threshold Ascending method as specified in ISO 82531:1.5. As no tympanometry or bone conduction audiology was done at the follow-up assessment, cases were categorized into children with “no signs of external or middle ear abnormalities” or “obvious signs of external or middle ear abnormalities” based on the otoscopic evaluation conducted by the Audiologist. “Obvious signs of external or middle ear abnormalities” included observations of occluding wax, otorrhoea or abnormal tympanic membrane. Criteria constituting hearing loss was pure tone average (PTA) (0.5 – 4 kHz) of 25 dB HL or greater in the worse ear [8]. Degree of hearing loss was largely based on the classification by the World Health Organization (26–40 dB HL being “mild”, 41–60 dB HL “moderate”, 61–80 dB HL “severe” and 81 dB HL or greater “profound”) [3]; 25 dB HL was included in the “mild” category.

Children who failed the initial vision screening were retested on the same day at their preschool by the trained CHWs, using the validated Peek Acuity application on the same smartphone (Peek Vision, London, United Kingdom) [26]. This test follows the standard Early Treatment Diabetic Retinopathy Study chart design, using a Tumbling E optotype, and is capable of acuity measurements consistent with test–retest variability of acuities measured using 5-letters-per line retro-illuminated LogMAR (logarithm of minimum angle of resolution) charts [25, 26]. Vision loss was indicated when the visual acuity was less than 0.3 LogMar in both eyes, or less than 0.4 LogMar in the worse eye. Degree of vision loss was categorized as “Mild” (0.4 LogMar), “Moderate” (0.5–0.9 LogMar), “Severe” (1–4 LogMar) and “No Response” (5 LogMar) [4]. Recent studies demonstrated that minimally trained non-specialist health workers (e.g. CHWs) are able to conduct screening services equivalent to that of professional healthcare workers, when equipped with mHealth technology [27, 28, 30].

#### Referrals after follow-up assessments

Children presenting with hearing or vision loss at the follow-up assessment were referred to public health care clinics (audiology or optometry clinics) in their area for further assessments and intervention [26, 32]. Children whose hearing was unable to be tested due to inconsistent and unreliable responses, were recorded on the database as “unable to test” and referred for evaluation at the health care clinics. Children who presented with “Normal” results (− 0.1–0.3 LogMar), but had obvious signs of ocular abnormality (such as strabismus or a teacher’s report of visual difficulty), were recorded on the database as “ocular morbidity” and referred for evaluation at the health care clinics. Parents of children who were referred were notified of the outcome via a letter and phone call [26]. A future study will report on the outcome of the clinic visits.

### Data storage and analysis

Data collected by the smartphone were uploaded to a cloud storage facility through mobile telephone networks at the end of each test [36, 37], using the mHealth Studio platform (hearX Group, South Africa) [26, 32]. The security of the mHealth app and server are provided by local data encryption at rest using Advanced Encryption Standard 256 bit [26].

Data of the follow-up test were extracted from the secure cloud-based server (mHealth Studio) to Microsoft Excel (2016) and coded according to test outcomes (sensory loss or not), characteristics (unilateral or bilateral) and severity of loss for descriptive statistical analysis. Logistic regression was used to estimate the association between the presence of sensory loss and gender and age using IBM SPSS Statistics for Windows (version 25.0 Armonk, NY). A *p*-value cut off was set at 0.05 and indicated the level of significance throughout this study.

## Results

**Fig. 1:**
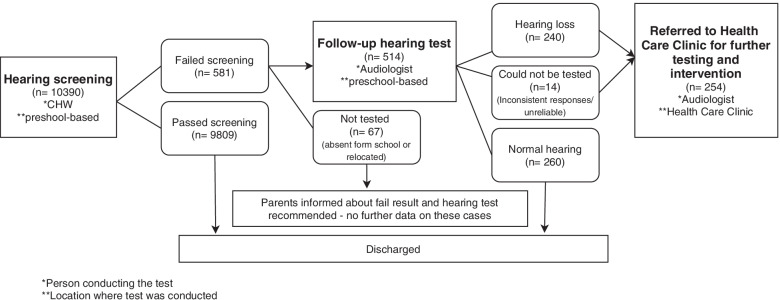
Flowchart of hearing screening program process and test personnel

**Fig. 2:**
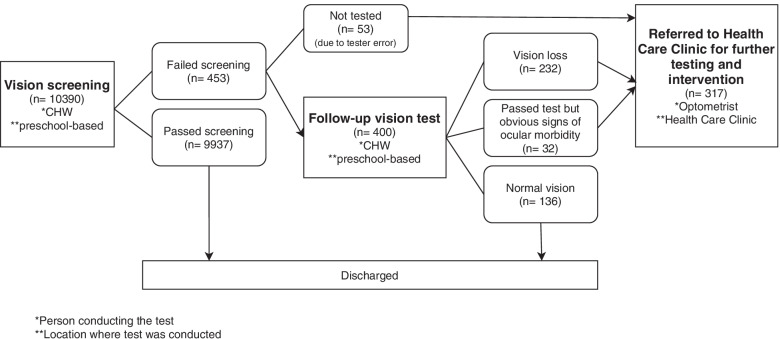
Flowchart of vision screening program process and test personnel

A total of 10,390 children (50.2% female) with a mean age of 5.7 years (SD 0.61) were screened at 298 preschools over 22 months (Figs. 1 and 2).

The overall screening referral rate was 5.6% (581 children) resulting from hearing screening (Fig. 1) and 4.4% (453) resulting from vision screening (Fig. 2). Seventy-two children (0.7%) failed both hearing and vision screening at the initial assessment.

### Follow-up hearing test

Follow-up hearing tests at the preschools were done on 88.5% (514) of children of whom 240 children (54.2% female) presented with hearing loss (Table 1 and Fig. 1). Fourteen of the children who failed the hearing screening and who were seen for a follow-up assessment, were unable to be tested due to inconsistent responses (Fig. 1). They were referred to the health care clinic for further tests, but they were not included as children with a hearing loss in this study. Half (260, 51%) of children presented with normal hearing at the follow-up hearing test and were discharged from the programme (Fig. 1). Prevalence for hearing loss at a PTA of 25 dB HL ranged between 2.3% (240/10390) (assuming none of the non-attenders and “unable to test” children had hearing loss) and 3.1% (321/10390) (assuming all the non-attenders and “unable to test” children presented with hearing loss). Of the 136 children with obvious signs of external and/or middle ear abnormalities, 95 (69.9%) had occluding wax and 41 (30.1%) had abnormal middle ear findings (Table 1).

**Table 1:** Characteristics of hearing loss across participants seen for follow-up by audiologist at preschools (*n* = 240)

*Characteristics*	*Bilateral % (n)*	*Unilateral % (n)*	*All losses % (n)*
**Hearing Loss**			
No signs of external or middle ear abnormality	64.4% (67/104)	35.6% (37/104)	43.3% (104/240)
External or middle ear abnormality	64.7% (88/136)	35.3% (48/136)	56.7% (136/240)
All hearing losses	64.6% (155/240)	35.4% (85/240)	100% (240/240)
**Degree of HL according to the worst ear**			
Mild (25–40 dB HL)	63.6% (84/132)	36.4% (48/132)	55.0% (132/240)
Moderate (41–60 dB HL)	66.3% (57/86)	33.7% (29/86)	35.8% (86/240)
Severe (61–80 dB HL)	27.3% (3/11)	72.7% (8/11)	4.6% (11/240)
Profound (81 dB HL or greater)	100% (11/11)	0	4.6% (11/240)

### Follow-up vision test

A follow-up vision test was done on 400 children (88.1%) on the same day that they failed the initial screening at the preschool (Fig. 2). A total of 232 children (46.1% female) had a vision impairment at the set criteria (Table 2), and a further 32 children passed the test but had obvious signs of ocular morbidity (Fig. 2). Prevalence of vision loss ranged between 2.2% (232/10390) (assuming none of the non-attenders had vision loss) and 2.8% (286/10390) (assuming all the non-attenders presented with vision loss). The laterality and degree of vision loss is shown in Table 2.

**Table 2:** Characteristics of vision loss across participants seen for follow-up vision test at preschools (*n* = 232)

*Characteristic*	*Bilateral % (n)*	*Unilateral % (n)*	*All losses % (n)*
**Vision Loss**			
All vision losses	59.1% (137/232)	40.9% (95/232)	100% (232/232)
**Degree of VL according to the worst eye**			
Mild (0.4 LogMar)	100% (11/11)	0	4.7% (11/232)
Moderate (0.5–0.9 LogMar)	56.2% (50/89)	43.8% (39/89)	38.4% (89/232)
Severe (1–4 LogMar)	66.7% (12/18)	33.3% (6/18)	7.8% (18/232)
No Response (5 LogMar)	56.1% (64/114)	43.9% (50/114)	49.1% (114/232)

Table 3 displays the prevalence of hearing loss and vision loss in the population of children screened at their preschools.

**Table 3:** Prevalence of sensory losses in the population of children screened at preschools (*n* = 10,390)

*Characteristics*	*All losses % (n)*	*Bilateral % (n)*	*Unilateral % (n)*
**Hearing Loss**			
No signs of external or middle ear abnormality	1.0% (104/10390)	0.6% (67/10390)	0.4% (37/10390)
External or middle ear abnormality	1.3% (136/10390)	0.8% (88/10390)	0.5% (48/10390)
All hearing losses	2.3% (240/10390)	1.5% (155/10390)	0.8% (85/10390)
**Degree of HL according to the worst ear**			
Mild (25–40 dB HL)	1.3% (132/10390)	0.8% (84/10390)	0.5% (48/10390)
Moderate (41–60 dB HL)	0.8% (86/10390)	0.5% (57/10390)	0.3% (29/10390)
Severe (61–80 dB HL)	0.1% (11/10390)	0.02% (3/10390)	0.08% (8/10390)
Profound (81 dB HL or greater)	0.1% (11/10390)	0.1% (11/10390)	0
**Vision Loss**			
All vision losses	2.2% (232/10390)	1.3% (137/10390)	0.9% (95/10390)
**Degree of VL according to the worst eye**			
Mild (0.4 LogMar)	0.1% (11/10390)	0.1% (11/10390)	0
Moderate (0.5–0.9 LogMar)	0.9% (89/10390)	0.5% (50/10390)	0.4% (39/10390)
Severe (1–4 LogMar)	0.2% (18/10390)	0.1% (12/10390)	0.1% (6/10390)
No Response (5 LogMar)	1.1% (114/10390)	0.6% (64/10390)	0.5% (50/10390)

Table 4 displays the distribution of sensory losses according to age and gender in children tested at their preschool. Logistic regression analysis found that age was a significant predictor of vision loss (*p* < 0.001), with each year older a participant was 51.4% less likely of having vision loss (OR: 0.49, 95% CI:0.39–0.60). Age was not a significant predictor of hearing loss (*p* > 0.05). Gender was not a significant predictor of hearing (p > 0.05) or vision loss (p > 0.05).

**Table 4:** Prevalence of sensory impairment according to age and gender

		Distribution of participants (n)	% of children with hearing loss (n)	% of children with vision loss (n)	% of children with combined sensory loss (n)
**Total**		100% (10390)	2.3% (240)	2.2% (232)	0.3% (27)
***Gender***	**Female**	50.2% (5215)	2.5% (130)	2.1% (107)	0.2% (12)
	**Male**	49.8% (5175)	2.1% (110)	2.4% (125)	0.3% (15)
***Age***	**4–5 years**	17.4% (1808)	2.5% (45)	3.7% (67)	0.5% (9)
	**5.1–6 years**	55.0% (5715)	2.4% (137)	2.4% (136)	0.3% (17)
	**6.1–7 years**	27.6% (2867)	2.0% (58)	1.0% (29)	0.03% (1)

## Discussion

This study aimed to estimate and describe hearing and vision loss among preschool children (4–7 years) in an underserved South African community. A critical issue in health services research related to infants and children is that of timely, necessary, and appropriate referrals for early childhood intervention services [1, 2, 16]. The development of mHealth has provided more opportunities for sensory screening at preschools in the community, to facilitate increased access to hearing and vision services. In this study, 5.6 and 4.4% of children failed the initial hearing and vision screen, respectively. These estimates compare well with previous studies reporting estimate referral rates of 5.6% for hearing [36] and 3.6% for vision [24]. Despite literature reporting that hearing and vision loss commonly co-occur [38, 39], only 0.7% of children failed both hearing and vision screening, indicating the value of offering dual sensory screening at the same time, as identifying an impairment in one modality does not predispose or preclude an impairment in the other [26]. This service-delivery model, where trained CHWs are utilized to screen both hearing and vision using the same smartphone, has been shown to be efficient and low-cost [24, 26].

A high proportion of the children who failed the screens completed the follow-up assessments (88.5% for hearing and 88.3% for vision). These figures are high compared to rates of 32.5 and 25.1% reported by Manus et al. (2020) and 45.3% reported by Hussein et al. (2018), when follow-up assessments were done at the health care facilities [23, 24]. Loss to follow-up after screening is widely reported as a barrier to healthcare [8, 23, 24, 27]. In previous studies, reasons for poor follow-up rates were attributed to transportation costs, leave of absence from work and long waiting periods at health care facilities [16, 23, 30]. The high follow-up rates of this study demonstrate the value of decentralized follow-up assessments conducted at the preschools in the community [26, 40]. In this study, the follow-up hearing tests were done by an audiologist. In low-resource settings, the availability and capacity of audiologists may pose a challenge to scaling up this model. For future implementation of such services, it is therefore proposed to enable CHWs to gather both threshold audiometric data and otoscopic images using a unified smartphone-based platform [21]. With smartphone-enabled otoscopes (smartphones coupled with specialized cameras allowing otoscopy to be utilized on the same platform), CHWs can easily capture images of the ear canal and tympanic membrane and save them to be shared and referenced in the future [21, 41]. The utilization of trained CHWs can further contribute to the affordability and the efficiency of the applied service-delivery model [21, 24, 26].

Out of the children who failed hearing and vision screening, 41.3% presented with hearing loss and 51.2% presented with vision loss at the follow-up assessment and were referred for treatment in the health care system. The community-based follow-up assessments assure selective referrals, thereby reducing the burden upon the health care systems and scarce specialized healthcare professionals [19, 21, 26].

Due to the risk of loss to follow-up at health care centres, it is more accurate to report the prevalence of sensory losses according to the follow-up assessments at the preschools at that point in time [23, 24]. The prevalence for hearing loss in this study ranged between 2.3 and 3.1%, depending on the assumptions for the proportion of non-respondents who were cases. Different criteria and testing methods and age cut-offs are used to determine sensory losses across studies, making it difficult to compare these prevalence estimates with the existing literature [23, 42]. The global prevalence of disabling hearing impairment (defined as PTA ≥ 35 dB HL in the better ear) among children 5–14 years of age was reported as 1.4% and prevalence in sub-Saharan Africa was 1.9% [10], whereas a study by Olusanya et al. (2020) reported global prevalence in 5–9 year olds for hearing loss as 3.8 and 4.5% for sub-Saharan Africa (criteria constituting hearing loss was PTA ≥ 20 dB HL in the better ear) [2]. Prevalence estimates have also been reported in preschool children in sub-Saharan Africa, ranging from 2.4% in Zimbabwe [43] and to 21.3% in Nigeria [44].

About half of the children with hearing loss (53.5%) had obvious signs of external and/or middle ear abnormalities. The prevalence of ear disease might have been even higher, as tympanometry was not conducted and therefore not all middle ear pathology was identified [8, 45]. The high prevalence of occluding wax and abnormal middle ear findings in the current study are in line with recent reports from the WHO, which postulates that the leading causes of childhood hearing loss in LMICs are conductive and treatable [46]. Studies have found conductive hearing loss to be the most common type of hearing loss found in preschool children in South Africa (65% in both studies) [23, 47]. This indicates a need for referral services in sub-Saharan Africa in order to ensure for appropriate treatment and follow-up service and highlights ear disease as a public health concern [22, 23, 47, 48].

The prevalence of bilateral hearing loss was found to be more common than that of unilateral hearing loss, in agreement with reports of others [23, 47]. Also in agreement with other studies, mild hearing loss was most prevalent, followed by moderate loss [23, 47]. This may be partially explained by impacted wax and otitis media and its sequelae [2, 10, 23, 47]. Early identification and appropriate management of both bilateral and unilateral hearing loss, as well as milder degree of hearing impairment are important since even a unilateral or mild hearing losses negatively affect educational outcome [2, 22, 46, 49]. Only 0.1% of children screened had a severe hearing loss and 0.1% of children had a profound hearing loss. In recent years, an increase of targeted hearing screening in Cape Town, South Africa, resulted in more children with permanent congenital or early-onset hearing loss (PCEHL) being identified and diagnosed at health care centres before the age of 4 years [47, 50]. Therefore, children with sensory losses between 4 to 7 years might already be enrolled into intervention programmes and preschools specifically for children with disabilities, thus excluding them from the prevalence reported in this study.

Prevalence of vision loss in the current study ranged between 2.2 and 2.8%. The global prevalence, as well as the prevalence in SSA, for 5–9 year olds are estimated at 3.1% for vision loss [2]*.* In comparison to previous studies, mild vision loss was least prevalent [2]. The severity of vision loss was based on degree of loss in the worst eye, possible contributing to the high prevalence of results indicating “No response” in this study.

More than half (137) of children with vision loss had bilateral loss and 95 had unilateral loss. Thirty-two children passed the visual acuity assessment, but had obvious ocular abnormality. Nirmalan (2003) found that CHWs can be trained effectively to identify children with ocular abnormalities and they should not be limited to screening for vision impairment alone [51]. Therefore, training of the CHWs should include identification of obvious signs of visual impairment (such as strabismus), in order for children who passed screening but present with abnormalities to also be referred for follow-up assessments and intervention. In LMICs, the majority of vision loss is either preventable or treatable [12, 16]. Therefore, early identification and intervention through vision screening is a priority within the WHO VISION 2020 (Right to Sight) programme [12].

It is reported that sensory impairments commonly co-occur, with an estimated 40 to 60% of children with hearing loss also having some degree of vision loss [38, 39]. In the population of children diagnosed with PCEHL at health care centres [47], there will most probably be a higher incidence of co-occurring sensory losses than the 0.3% of children found to have combined sensory losses in this study. Another consideration is that early childhood education is not compulsory in South Africa and it is possible that not all young children with sensory deficits attended preschool facilities targeted in this study [23, 26].

In agreement with previous studies, gender did not have a significant effect on sensory losses [22, 23, 36]. Age was a predicting factor of vision loss, however, the strength of the correlation was poor. The higher prevalence of vision loss in younger children might be ascribed to younger children not yet being enrolled in special schools or receiving treatment elsewhere. Other studies also showed no association between hearing impairment and age [22, 23].

### Strengths and limitations

Strengths of this study include a large study population, assessment of both hearing and vision, as well as the use of validated tools for community-based screening and assessments. Limitations of the current study included that sensitivity and specificity for these assessments could not be determined. The hearing assessment protocol did not include tympanometry or bone conduction audiometry and therefore, the nature (conductive versus sensori neural versus mixed hearing loss) and cause of hearing loss could not be determined. The visual assessment protocol did not include a basic ocular examination using torchlight and may have resulted in an underestimation of ocular morbidity. The cause of vision loss was also not determined.

## Conclusions

According to this study, hearing loss is prevalent in at least 22 per 1000 and vision loss is prevalent in at least 23 per 1000 preschool children in an underserved South African community*.* Community-based follow-up assessments ensured a high follow-up return rate (88.5% for hearing and 88.3% for vision) and assured selective referrals, thereby reducing the burden upon the health care systems and scarce specialized healthcare professionals. Children who were identified with sensory losses were referred to health care clinics where they received interventions (e.g. medical management, hearing aids or spectacles). Future studies aim to report on causes of visual or hearing loss, as well as outcomes and the impact of interventions on the children diagnosed with sensory impairments. Timely identification of sensory losses is essential to ensure optimal outcomes and can be facilitated through community-based hearing and vision services supported by mHealth technology.

## Data Availability

The dataset used and analysed during the current study are available from the corresponding author on reasonable request.

## Abbreviations

CHWs: Community Health Workers
dB HL: Decibel hearing level
kHz: Kilohertz
LMICs: Low-and middle income countries
PCEHL: Permanent congenital early-onset hearing loss
PTA: Pure tone average
SDGs: Sustainable development goals
SSA: Sub-saharan Africa
WHO: World Health Organization
